# The Role of TLR4 Asp299Gly and TLR4 Thr399Ile Polymorphisms in the Pathogenesis of Urinary Tract Infections: First Evaluation in Infants and Children of Greek Origin

**DOI:** 10.1155/2019/6503832

**Published:** 2019-04-30

**Authors:** Panagiota Karananou, Despoina Tramma, Socrates Katafigiotis, Anastasia Alataki, Alexandros Lambropoulos, Efimia Papadopoulou-Alataki

**Affiliations:** ^1^4th Department of Pediatrics, Aristotle University of Thessaloniki, School of Medicine, Papageorgiou General Hospital, Ring Road Nea Efkarpia, 56403 Thessaloniki, Greece; ^2^Molecular Biology Laboratory, 1st Department of Obstetrics and Gynecology, Aristotle University of Thessaloniki, School of Medicine, Papageorgiou General Hospital, Ring Road Nea Efkarpia, 56403 Thessaloniki, Greece

## Abstract

Urinary tract infections are one of the most common and serious bacterial infections in a pediatric population. So far, they have mainly been related to age, gender, ethnicity, socioeconomic level, and the presence of underlying anatomical or functional, congenital, or acquired abnormalities. Recently, both innate and adaptive immunities and their interaction in the pathogenesis and the development of UTIs have been studied. The aim of this study was to assess the role and the effect of the two most frequent polymorphisms of TLR4 Asp299Gly and Thr399Ile on the development of UTIs in infants and children of Greek origin. We studied 51 infants and children with at least one episode of acute urinary tract infection and 109 healthy infants and children. We found that 27.5% of patients and 8.26% of healthy children carried the heterozygote genotype for TLR4 Asp299Gly. TLR4 Thr399Ile polymorphism was found to be higher in healthy children and lower in the patient group. No homozygosity for both studied polymorphisms was detected in our patients. In the group of healthy children, a homozygote genotype for TLR4 Asp299Gly (G/G) as well as for TLR4 Thr399Ile (T/T) was showed (1.84% and 0.92 respectively). These results indicate the role of TLR4 polymorphism as a genetic risk for the development of UTIs in infants and children of Greek origin.

## 1. Introduction

Urinary tract infections (UTIs) are one of the most common and potentially important and serious bacterial infections in a pediatric population affecting approximately 2.6-7.5% of febrile children annually [1]. Up to 11% of girls and 7% of boys will experience UTI by the age of 16 years, and the recurrence of the infection seems to happen very often (13–19%) [2, 3]. Although the prognosis of a single episode of febrile UTI is usually good, major concerns are related to the risk of permanent renal injury, which may predispose to hypertension and renal insufficiency in some patients [4, 5]. Identifying children at major risk could therefore allow for the implementation of preventive measures to preserve the final renal outcome in these children.

In the past, UTIs have mainly been related to age, gender, ethnicity, socioeconomic level, and the presence of underlying anatomical or functional, congenital, or acquired abnormalities. Recently, the function of innate immunity in the control of UTIs was studied. The need to clarify the role of the host (uroepithelium) response to recognize uropathogenic strains and the local immune response against their invasion has made the study of new risk factors imperative. The discovery of Pattern Recognition Receptors (PRRs) introduces the research of the role of innate immunity in relation to UTIs. PRRs are important immunologic biosensors that detect pathogens within the host's cells and tissues by recognizing their structural components (PAMPS) [6, 7].

Toll-like receptors (TLRs) are the best studied PRRs. Among them, 13 TLR family members recognize products of a variety of invading antigens. The TLR4 is a 224 amino acid protein that is encoded by the TLR4 gene in humans (Gene ID = 7099; 9q33.1), which spans a genomic region of ~13.3 kb with three exons (NCBI; https://www.ncbi.nlm.nih.gov/). TLR4 is widely distributed on the cell surface of many cells, mainly monocytes, neutrophils, and epithelial cells such as those of uroepithelium. Several simple nucleotide polymorphisms (SNPs) have been identified in the TLR4 gene with some of them being strongly associated with an increased susceptibility to Gram-negative bacterial infections and an increased incidence of septicemia [8].

The two most studied SNPs of TLR4 in animal models and in vitro studies are TLR4 Asp299Gly and TLR4 Thr399Ile. Specifically, in the Asp299Gly polymorphism, we have an A to G transition (SNP ID = rs4986790), resulting in aspartate-glycine substitution at position 299 and, in the TLR4Thr399Ile polymorphism, a C to T transition (SNP ID = rs4986791), resulting in threonine-isoleucine substitution at position 399 [9, 10]. These two nonsynonymous SNPs have been described with population frequencies > 5%. Τhey are identified in human populations more often compared to most of other SNPs with low frequencies (<1%) [11]. Therefore, the study of TLR4 Asp299Gly and TLR4 Thr399Ile polymorphisms is of enormous clinical and therapeutic interest, as they could provide new aspects and more efficient approaches in the treatment and prevention of UTIs in children. The aim of this study was to conduct a genetic study of the TLR4 Asp299Gly and TLR4 Thr399Ile polymorphisms in a population of infants and children of Greek origin and to assess their role and relation to UTIs. The secondary aim was to compare demographic, laboratory, and clinical parameters between patients, carriers, and noncarriers of the polymorphisms.

## 2. Materials and Methods

### 2.1. Study Design and Populations

This is a case-control study in a population of Greek origin. In total, 160 infants and children were included. We studied 51 infants and children (19 males, 32 females) with at least one episode of acute urinary tract infection (AUTI) in their records, with or without underlying anatomic genitourinary anomalies (average age at entry to the study is 3.27 ± 3.20). We also studied 109 phenotypically healthy infants and children (average age at entry to the study is 6.65 ± 4.57). The latter group of children had no history of severe or chronic illness and of UTI, no symptoms of acute infection, and negative urine culture at the time of sample collection.

All patients who participated in the study should meet the following criteria: (a) Those suffering from lower urinary tract infection (cystitis) should have significant bacteriuria of a single microorganism (10^5^ CFU mL^−1^), erythrocyte sedimentation rate (ESR) and C-reactive protein (CRP) at normal levels (ESR < 20 mm/h and CRP < 0.8 mg/dL, respectively), symptoms of dysuria, polyuria and/or abdominal pain, and absence of fever [12, 13]. (b) Those suffering from upper urinary tract infection (pyelonephritis) should have a urine white blood cell count of ≥25 cells per *μ*L (1+ with a dipstick) and significant bacteriuria of a single microorganism (10^5^ CFU mL^−1^), plus the presence of ≥2 of the following criteria: fever ≥ 38°C, high levels of ESR and/or CRP or neutrophil levels above normal values for age [10, 11].

Patients formed Group A which was subsequently divided in two subgroups: Group A1 (35/51 patients)—infants and children diagnosed with lower UTI, and Group A2 (16/51 patients)—infants and children diagnosed with upper UTI. All patients were hospitalized at the Pediatric Clinic, Fourth Department of Pediatrics, Faculty of Medicine, Papageorgiou General Hospital, Aristotle University of Thessaloniki. The control group (Group C) consisted of healthy infants and children who were followed up at the General Pediatrics Outpatient Unit of the Fourth Department of Pediatrics.

In all patients and 79/109 of Group C, personal history was recorded, a detailed clinical examination was conducted, and a blood sample was collected for the determination of biochemical and immunological parameters as well as a urine sample. They had complete blood count, measurement of urea and creatinine, ESR, CRP, urine culture, and urine microbiological analysis. A complete immunological profile was also evaluated in every child including immunoglobulins: IgG IgA, IgM, IgG subclasses (IgG_1_, IgG_2_, IgG_3_, IgG_4_), and complement components C3 and C4. In all patients, kidney ultrasound was performed. In all 160 children of the study, genetic testing was performed to determine the TLR4 Asp299Gly and TLR4 Thr399Ile polymorphisms. Before entering the study, an informed consent was obtained by parents or guardians of the children. The research protocol was declared at the service of ClinicalTrials.gov and approved by the Ethics Committee of the Faculty of Medicine of Aristotle University of Thessaloniki. The study was conducted according to the criteria of the Declaration of Helsinki.

### 2.2. TLR4 Asp299Gly and Thr399Ile Genotyping

#### 2.2.1. Genomic Isolation of Deoxyribonucleic Acid (DNA)

Whole blood was collected into a sterile vacutainer containing anticoagulant ethylenediaminetetraacetic acid (EDTA). Molecular genetic analysis was carried out on genomic DNA extracted from EDTA using according to the manufacturer's instructions.

#### 2.2.2. Genomic DNA Isolation and Polymerase Chain Reaction (PCR)

Genomic DNA was extracted from the whole blood using Invisorb Spin Blood Mini Kit (STRATEC Molecular GmbH, Germany) following the manufacturer's instructions. The 249 bp gene fragment carrying the TLR4 Asp299Gly mutation was amplified from whole genomic DNA using the 5′-GATTAGCATACTTAGACTACTACCTCCATG-3′ and 5′-GATCAACTTCTGAAAAGCATTCCCAC-3 forward and reverse primers, respectively, and the 407 bp fragment carrying the TLR4 Thr399Ile mutation was amplified using the 5′-GGTTGCTGTTCTCAAAGTGATTTTGGGAGAA-3′ and 5′-ACCTGAAGACTGGAGAGTGAGTTAAATGCT3′ forward and reverse primers, respectively. PCR primers were designed to allow a distinction of wild-type and mutant TLR4 alleles based on the presence of restriction enzyme recognition sites. PCR was performed as follows: 200-300 ng of genomic DNA, 1.5 mM MgCl_2_, 400 mM dNTPs, 20 pM forward primer, 20 pM reverse primer, and 1.5 U Taq polymerase (Ampli-Taq, Applied Biosystems, Foster City, CA, USA). Cycling conditions were as follows: initial denaturation at 95°C for 5 minutes, followed by 35 cycles of 30 seconds at 95°C, 30 seconds at 51°C and 45 seconds at 72°C, and final extension at 72°C for 10 minutes. All PCR reactions were carried out in a Prime Thermal Cycler (Techne, Stone, Staffordshire, UK). PCR products were visualized in a 2% agarose gel.

#### 2.2.3. Restriction Fragment Length Polymorphism Method (RFLP)

The presence of TLR4 gene mutations was determined by RFLP. The 249 bp PCR product was cut with NcoI to determine the presence of TLR4 Asp299Gly mutation, and the 407 bp PCR product was cut with HinfI to determine for the presence of Thr399Ile mutation, as described by Lorenz et al. [14]. Restriction digest products were visualized on a 3.5% agarose gel.

#### 2.2.4. Electrophoresis

Subjects were subjected to electrophoresis on gels containing a mixture of 3% and 3.5% agarose gel.

### 2.3. Statistical Analysis

Data were analyzed using the IBM statistical program Statistical Package for Social Science software for Windows, version 22.0 (SPSS Inc., Chicago, Illinois, USA). Expected and observed frequencies of genotypes and alleles in patients and controls were compared in 2 × 3 and 2 × 2 tables, respectively, according to the Hardy-Weinberg equilibrium. The differences in frequencies of genotypes and alleles between patients and controls and between patient subgroups based on qualitative variables were analyzed by the *χ*^2^ or Fisher exact test. Normal distribution of quantitative variables was examined using the Kolmogorov-Smirnov test. Normally distributed mean age at diagnosis was analyzed according to genotype analysis using the analysis of variance (ANOVA). The level of statistical significance was defined at *p* < 0.05.

## 3. Results

### 3.1. Demographic and Clinical Features of the Study Groups

The demographic and the clinical features of Group A (patients), Subgroups A1 and A2 (patients), and Group C (controls) are shown in Tables 1 and 2. Fifty-one patients were evaluated. Thirty-five patients were diagnosed with lower UTI and sixteen with upper UTI. For forty-one patients, that was their first episode of UTI while ten patients suffered from UTI twice or more. Girls developed UTI more frequently than boys. Most of the episodes of the UTIs concerned the lower urinary tract in equal proportion between boys and girls. Furthermore, girls developed upper UTI more often compared to boys.

Urine cultures of all patients showed Gram-negative bacteria with *E. coli* superiority in 43/51 patients (84.3%). The renal ultrasound revealed that 13/51 of the examined patients (25.5%) had pathological findings.

### 3.2. Laboratory Data of the Study Groups

Laboratory data are shown in Table 3. The total number of the leukocytes, the absolute count of the neutrophils, and the inflammatory markers (ESR, CRP) were indicatively increased in patients than in healthy children (79/109), as expected. Finally, from the analysis of the immunological parameters (immunoglobulins IgG, IgA, and IgM and subclasses of IgG), no primary immunodeficiency was detected in any of the study subjects.

### 3.3. Study of TLR4 Polymorphisms

In this study, the distribution of genotypes in both patients and healthy subjects did not differ significantly from the expected Hardy-Weinberg equilibrium (*p* = 0.987 and *p* = 0.959, respectively). The TLR4 Asp299Gly genotype was detected more frequently in patients (27.5%) compared to controls (10.1%) at a statistically significant level (*p* = 0.02) (Table 4). The TLR4 Thr399Ile genotype was more frequent in the control group (11.93%) than in the patient group (1.96%), but at a nonstatistically significant level (*p* = 0.34) (Table 4). The copredominant model of inheritance emerged for the TLR4 Asp299Gly genotype and the predominant model for the TLR4 Thr399Ile genotype, as the most probable reaching a statistical significance (*p* < 0.05).

The distribution of TLR4 Asp299Gly and TLR4 Thr399Ile genotypes carrying the predisposing allele did not differ significantly between males and females in the patient group as well as compared with the control group (Table 5). No statistically significant association of the TLR4 Asp299Gly and TLR4 Thr399Ile polymorphisms as far as the number of the episodes of the UTIs in any of the subgroups of patients (A1, A2) was found (Table 6).

Although 13/51 (25.5%) of the examined patients had abnormal kidney ultrasound findings, only 3 patients heterozygous for the TLR4 Asp299Gly polymorphism were among them. Therefore, no statistically significant association of the TLR4 Asp299Gly polymorphism with abnormal findings in kidney ultrasound was found (Table 6). However, the limitation of this comparison is the small number of the patients' groups examined. No statistically significant association was found between the studied polymorphisms and the total number of leukocytes, the percentage of neutrophils and lymphocytes, the inflammatory markers (ESR, CRP), and the values of urea and creatinine; IgG, IgA, and IgM immunoglobulins; the subclasses of IgG; and the complements C3 and C4 (Tables 7 and 8).

## 4. Discussion

Urinary tract infection is one of the most usual bacterial infections in infancy and childhood that occurs in 8.4% of girls and 1.7% of boys under the age of 6 years [15]. The most commonly isolated organism in infants and children with urinary tract infections is by far *E. coli* with a high rate of 80-90% followed by *Enterococcus* species, *Enterobacter*, *Pseudomonas aeruginosa*, *Klebsiella pneumonia*, *Proteus mirabilis*, and *Staphylococcus spp.* [16].

The urinary tract is continuously exposed to microorganisms and pathogens mainly coming from the intestinal tract. Ιt resists by developing defense mechanisms that represent mainly functions of innate immunity. Uroepithelium arises barriers, especially when it encounters infections of the lower urinary tract. For this reason, the role of innate immunity is the best studied regarding the pathogenesis of urinary tract infections compared to that of adaptive immunity.

Over the last 20 years, researchers have studied both innate and adaptive immunities and their interaction in the pathogenesis and the development of UTIs. It is suggested that genetic factors are capable of interfering with any step during the process of bacteria invasion and regulate the inflammatory response before, during, and after UTI. These factors are different gene products such as cytokines, receptors, and adhesion molecules. TLRs are receptors expressed either on the surface of many cells in the blood or on the surface of epithelial cells, such as uroepithelium. They play a pivotal role in the identification of infectious factors and the rapid activation of signaling pathways for the elimination of microbial pathogens or the mobilization of adaptive immunity. It is perceived that TLRs are acting as a connecting bridge between innate and adaptive immunities. In the last 10 years, researchers have focused on polymorphisms or polymorphism combinations of genes coding for TLRs [7, 17].

The aim of this study was to assess the role and the effect of the two most frequent polymorphisms of TLR4 Asp299Gly and Thr399Ile on the development of UTIs in infants and children of Greek origin. Identifying the possible role of genetic variants in UTIs would help clinicians understand the pathogenesis of the inflammation and distinguish which children have a greater risk of developing UTI, based on their genetics.

The association of TLR4 Asp299Gly or Thr399Ile polymorphisms with a faulty response to the lipopolysaccharide (LPS) of Gram-negative bacteria in mice was first reported by Arbour et al. in 2000. They suggested that these mutations of the TLR gene may affect the TLR structure or expression and therefore have a negative effect on the response to bacterial endotoxins. This was later confirmed by many other studies [11, 18–23].

Case-control studies, so far, have established associations of TLR4 Asp299Gly or Thr399Ile polymorphisms with the development of a variety of diseases. Most of the studies were conducted on adult populations.

The first reference to the relationship between TLR4 and UTIs is found in 2003 by Schilling et al., who demonstrated that TLR4 on stromal and hematopoietic cells, in mice, mediates innate resistance to uropathogenic *E. coli.* This study is one of the first ones to reveal that bladder epithelial cells play a critical role in TLR4-mediated innate immunity *in vivo* during a mucosal bacterial infection [24].

Since 2006, however, numerous and continuously growing studies on human populations have been done dealing with TLR4 polymorphisms and their association with UTIs [7, 11, 25–31]. As far as the study of all TLR polymorphisms and especially of TLR4 polymorphisms in pediatric populations is concerned, the international literature can present only a limited number of articles. Moreover, these studies are heterogeneous regarding the ethnicity of the population being studied and thus difficult to compare with each other and come to a general agreement. What is worth mentioning is that the relationship of TLR4 Asp299Gly and TLR4 Thr399Gly polymorphisms to UTIs in infants and children in Greece has never been studied so far.

Reviewing the literature, we found that the first to study a possible relation of TLR4 polymorphisms with UTIs were Karoly et al. [32], in Hungary. In this study, an attempt was made to assess among other parameters the role of TLR4 A(896)G polymorphisms using allele-specific PCR in 103 children with recurrent UTI and to compare the allelic prevalence with reference values of 235 healthy children. TLR4 299AG genotype and TLR4 299G alleles were observed with an increased frequency statistically significant in children with recurrent episodes of UTI compared to the control group. It was also found that the TLR4 299AG genotype and the TLR4 299G alleles were also observed more frequently in children with recurrent UTI without vesicoureteral reflux (VUR) than in children with VUR. This finding is in accordance with our finding that notes that TLR4 A/G genotype is a risk factor for recurrent UTI independent of urinary anomalies [32].

In our results, we found that 27.5% of patients and 8026% of healthy children carried the heterozygote genotype for TLR4 Asp299Gly, a difference which was statistically significant. On the opposite, TLR4 Thr399Ile polymorphism was higher in healthy children (11.01%) and lower in the patient group (1.96%). No homozygosity for both studied polymorphisms was detected in our patients. However, in the group of healthy children in our study, 1.84% showed a homozygote genotype for TLR4 Asp299Gly (G/G) and 0.92% showed a homozygote genotype for TLR4 Thr399Ile (T/T).

Mutlubas et al. [33] tried to investigate the distribution of TLR4 gene polymorphisms among 69 pediatric renal-transplanted patients in relation to chronic allograft nephropathy and 115 healthy controls. Neither renal recipients nor healthy controls showed a homozygote genotype for Asp299Gly and Thr399Ile polymorphisms [33]. According to Mutlubas et al., as far as the heterozygote genotype for TLR4 Asp299Gly is concerned, 6 healthy children were found to carry it (5.3%). This percentage is lower compared to our finding (8.26%). As far as the TLR4 Thr399Gly polymorphism is concerned, a percentage (4.3%) lower than that of our study (11,01%) in 5 of the healthy children was found. Our findings regarding the percentage of both the genotype of the TLR4 Asp299Gly polymorphism and its alleles and the part of the urinary tract infected were in agreement with the study of Yin et al. which showed that the gene prevalence of TLR4c.896A<G and TLR4 896 G alleles in adult patients with lower UTI (urethritis, cystitis) was higher than that in the control group [28]. On the contrary, Hawn et al. [34] showed an opposite effect compared to our study suggesting that TLR4c.896A<G polymorphism is associated with a protective role against recurrent UTI and cystitis, but not for pyelonephritis (PN) in a study population of adult women [34]. In the study of Ertan et al. [35], in children with rUTI, TLR4 gene Thr399Ile polymorphism was not observed in any child, a finding that comes in disagreement with our study where TLR4 gene Thr399Ile polymorphism was found in 1.96% of our patients and 11.01% of the healthy children (in heterozygosity). Genotype distribution and allele frequency of Asp299Gly polymorphism were similar both in the children with rUTI and in healthy controls; thus, this study could not reveal a significant role of this gene in the pathogenesis of UTI [35]. Akil et al. [13] found out that TLR4 Asp299Gly polymorphism and TLR-4 (896) G allele frequency were not different in children with UTI in comparison with healthy children (genotype frequency 12.5 vs. 15.1% and allele frequency 7.1 vs. 6.9%, respectively). Our study however showed a statistical significant difference between the genotype frequencies of the TLR4 Asp299Gly polymorphism which was found higher in patients than in healthy children. This study also pointed out a possible association between genetic factors and renal scar formation. Although no significant difference in TLR4 Asp299Gly polymorphism was found between scar-positive and scar-negative pyelonephritis patients, it was nearly two times more in scar-positive pyelonephritis [13]. In Turkey, Bayram et al. [17] tried to determine the relation of TLR-4 Asp299Gly and Thr399Ile polymorphisms to febrile UTI and renal scar development in children. In the group of children with scars, the incidence rate of TLR4 Asp299Gly and TLR4 Thr399Ile polymorphisms was found at 3.5% and at 14% while in the group of children without scars at 7% and at 12.2% respectively. These results contradict ours, since we found TLR4 Thr399Gly polymorphism is rare in children with UTI, whereas TLR4 Asp299Gly polymorphism is more common [17]. Recently, Harshman et al. [36] investigated the prevalence of the TLR4 polymorphism (Asp299Gly, Gly299Gly) among children with chronic pyelonephritis and healthy children. In the majority of healthy children (96.8%), the normal distribution of alleles AA of TLR4 gene was detected. AG heterozygous genotype was detected in a nonsignificant percentage of 3.2% and mutant genotype GG was not detected at all. These findings differ from ours as we found, in the group of healthy children, a higher percentage of the TLR4 Asp299Gly polymorphism in heterozygosity (8.26%) as well as in homozygosity. In children with chronic pyelonephritis, AG heterozygous genotype was detected at a 86.7% frequency and mutant genotype GG at a 11.7% frequency respectively, and it reliably exceeded the parameters of the control group. These results differ once more with those of our study where in the patient group, no child with mutant GG genotype was found and a frequency of 27.5% was detected for AG heterozygous genotype [36]. In the study of Hussein et al. [37], it was showed that 88.9% of the children with UTIs and 94% of the healthy children did not carry the TLR4 Asp299Gly polymorphism at all. 10% of the patients' group and 6% of the healthy group carry the AG heterozygous genotype for the TLR4 Asp299Gly polymorphism. Only 4 patients (1.1%) carried the mutant genotype G. In addition, they showed for the TLR4 Thr399Ile polymorphism that the genotype and the allele frequency did not differ significantly between the patients and the control group. The causative role of the TLR4 Asp299Gly was suggested in the occurrence and progression of UTIS to involve renal parenchyma, which is in total agreement with the results of our study. Moreover, the percentage of TLR4 Asp299Gly (A/G) polymorphism in our patients was nearly three times higher than that reported by Hussein et al. (27.5% vs. 10%, respectively) which reinforces the hypothesis that TLR4 Asp299Gly polymorphism is related to the risk of developing UTI [37].

In this study, we made an attempt to record the frequency of TLR4 Asp299Gly and TLR4 Thr399Ile polymorphisms in healthy children and in children with one or more episodes of UTI. Moreover, we tried to look into a possible relation between the presence of these polymorphisms with parameters from their personal history such as gender, number of UTI episodes, renal function, kidney ultrasound findings, and laboratory data. What differentiates our study is that the percentage of the heterozygous for the TLR4 Asp299Gly polymorphism patients was much higher than any other percentage recorded in similar studies so far. TLR4 Thr399Ile polymorphism was also found to be more frequent in healthy children. Our results suggest a causative role of the TLR4 Asp299Gly polymorphism in the occurrence and progression of UTIs in children of Greek origin while, on the contrary, a protective role of the TLR4 Thr399Ile polymorphism against urinary tract infections. These findings could be attributed to the fact that the distribution of many SNPs differs among different populations. It should also be taken into consideration the fact that these results may be applicable in the Greek children and not in the general population. More and larger studies need to be conducted to confirm this statement.

## 5. Conclusions

Our data is the first to be published in Greece, addressing the role of TLR4 Asp299Gly and TLR4 Thr399Gly polymorphisms as a genetic risk for the development of UTIs in infants and children of Greek origin. We identify the possible role of the TLR4 genetic variants in the pathogenesis of UTI that would help to early recognition of the children at a greater risk and could serve as a useful tool to personalized management of UTIs in children. Our study contributes to growing evidence of the role of TLRs to UTI, however indicating the need for a larger sample and more multicenter studies.

## Figures and Tables

**Table 1:** Demographic data of the children of the study.

	Group Α (patients)	Group Α1	Group Α2	Group C (controls)
*n*	51	35	16	109
Age (years) Mean ± SD	3.27 ± 3.20	3.73 ± 3.70	2.34 ± 2.12	6.65 ± 4.57
*p*				0.001
Males	19	17	2	55
Females	32	18	14	54
*p*				0.017

**Table 2:** Clinical data of the patients.

	Group A (patients)	Gender (M/F)	
	Total (*n* = 51)	Females (*n* = 32)	Males (*n* = 19)
Lower UTI (Group Α1)	35	18	17
Upper UTI (Group Α2)	16	14	2
1 episode of UTI	41	28	13
≥2 episodes of UTI	10	4	6
Gram-/Gram+	51/0	32/0	19/0
US (+/-)	13/38	7/25	6/13

UTI: urinary tract infection; US: ultrasound.

**Table 3:** Laboratory data of the study groups.

Parameters	Patients (*N* = 51)	Controls (*N* = 79)	*p* (*t*-test)
	Mean ± SD	Mean ± SD	
Leukocytes (×10^3^/u)	14621 ± 5761	9308 ± 2719	<0.001
Neutrophils (%)	55.8 ± 16.38	49.9 ± 15.71	<0.001
Lymphocytes (%)	31.7 ± 14.73	37.54 ± 13.86	<0.001
Absolute neutrophil count	8270 ± 4598	4657 ± 2278	<0.001
IgG (g/L)	7.03 ± 3.38	10.07 ± 2.55	<0.001
IgA (g/L)	0.60 ± 0.50	1.11 ± 0.71	<0.001
IgM (g/L)	0.91 ± 0.47	1.15 ± 1.12	0.419
IgG1 (g/L)	4.91 ± 2.69	6.84 ± 1.91	<0.001
IgG2 (g/L)	1.30 ± 0.83	2.13 ± 1.32	<0.001
IgG3 (g/L)	0.29 ± 0.17	0.41 ± 0.18	<0.001
IgG4 (g/L)	0.40 ± 0.40	1.12 ± 0.96	<0.001
C3 (g/L)	1.42 ± 0.62	1.14 ± 0.24	<0.001
C4 (g/L)	0.23 ± 0.10	0.18 ± 0.50	0.004
CRP (mg/dL)	6.36 ± 6.26	0.50 ± 0.42	<0.001
ESR (mm)	45.75 ± 35.09	9.57 ± 6.41	<0.001
Serum urea (mg/dL)	22.29 ± 6.94	27.27 ± 7.18	<0.001
Serum creatinine (mg/dL)	0.47 ± 0.09	0.54 ± 0.099	<0.001

**Table 4:** The distribution of TLR4 Asp299Gly and Thr399Ile polymorphisms in patients and controls.

	Patients		Controls	
Frequency (%)				
	Group A (*N* = 51)	Group A1 (*N* = 35)	Group A2 (*N* = 16)	Group C (*N* = 109)
TLR4 Asp299Gly	Genotype			
AA	37 (72.5)	25 (71.4)	12 (75.0)	98 (89.9)
AG	14 (27.5)	10 (28.6)	4 (25.0)	9 (8.26)
GG	0 (0.00)	0 (0.00)	0 (0.00)	2 (1.84)
*p*		0.02446		
*Allele*				
A	88 (0.86)	60 (0.86)	28 (0.88)	205 (0.94)
G	14 (0.14)	10 (0.14)	4 (0.12)	13 (0.06)
*p*		0.06361		
TLR4 Thr399Ile	Genotype			
CC	50 (98.04)	34 (97.1)	16 (100)	96 (88.07)
CT	1 (1.96)	1 (2.9)	0 (0.00)	12 (11.01)
TT	0 (0.00)	0 (0.00)	0 (0.00)	1 (0.92)
*p*		0.3465		
*Allele*				
C	101 (0.99)	69 (0.99)	32 (1)	(0.94)
T	1 (0.01)	1 (0.01)	0 (0.00)	(0.06)
*p*	0.542			

**Table 5:** The distribution of TLR4 Asp299Gly and TLR4 Thr399Ile polymorphisms in patients and controls according to gender.

Gender *n* (%)									
SNPs	Genotype	Group Α (*N* = 51)		Group Α1 (*N* = 35)		Group Α2 (*N* = 16)		Group C (*N* = 109)	
		Males	Females	Males	Females	Males	Females	Males	Females
TLR4 Asp299Gly	AA	15 (79)	22 (69)	14 (82)	12 (67)	1 (50)	10 (71)	50 (90)	48 (89)
	AG + GG	4 (21)	10 (31)	3 (18)	6 (33)	1 (50)	4 (29)	5 (10)	6 (11)
	*p*	0.4301		0.2886		0.5408		0.7263	
TLR4 Thr399Ile	CC	18 (95)	32 (100)	16 (94)	18 (100)	2 (100)	14 (100)	51 (93)	45 (83)
	CT + TT	1 (5)	0	1 (6)	0	0	0	4 (7)	9 (17)
	*p*	0.19		0.2965		NA		0.1303	

**Table 6:** Analysis of the relation between genotypes and the number of urinary tract infections and findings of the ultrasound.

	Number of episodes						
SNPs	Genotypes	Group Α (*N* = 51)		Group Α1 (*N* = 35)		Group Α2 (*N* = 16)	
		1	≥2	1	≥2	1	≥2

TLR4 Asp299Gly	AA	29 (70.7)	7 (70)	19 (73)	6 (66.7)	11 (73.3)	2 (100)
	AG + GG	12 (29.3)	3 (30)	7 (27)	3 (33.3)	4 (26.7)	0 (0)
	*p*	0.9637		0.713		0.4036	
TLR4 Thr399Ile	CC	40 (97.6)	10 (100)	25 (96.2)	9 (100)	15 (100)	1 (100)
	CT + TT	1 (2.4)	0 (0)	1 (3.8)	0 (0)	0 (0)	0 (0)
	*p*	0.6179		0.5505		NA	

	Findings of the ultrasound						
SNPs	Genotypes	Group Α (*N* = 51)		Group Α1 (*N* = 35)		Group Α2 (*N* = 16)	
		Normal (%)	Pathological (%)	Normal (%)	Pathological (%)	Normal (%)	Pathological (%)

TLR4 Asp299Gly	AA	27 (71)	10 (77)	19 (73)	6 (66.7)	8 (66.7)	4 (100)
	AG + GG	11 (29)	3 (23)	7 (27)	3 (33.3)	4 (33.3)	0 (0)
	*p*	0.4961		0.7137		0.1824	
TLR4 Thr399Ile	CC	37 (97.3)	13 (100)	25 (96.1)	8 (100)	12 (100)	4 (100)
	CT+TT	1 (2.7)	0 (0)	1 (3.9)	0 (0)	0 (0)	0 (0)
	*p*	0.5547		0.5734		NA	

**Table 7:** Analysis of the relation between genotypes and WBC, neutrophils, lymphocytes, ESR, CRP, serum urea, and serum creatinine.

SNPs	Genotypes	Mean ± SD (*n*)						
		WBC	Neutrophils (%)	Lymphocytes (%)	ESR (mm)	CRP (mg/dL)	Serum urea (mg/dL)	Serum creatinine (mg/dL)
TLR4 Asp299Gly	AA	15165 ± 5986	54.84 ± 17.19	32.62 ± 15.13	48.97 ± 34.13	6.44 ± 5.94	21.95 ± 7.29	0.46 ± 0.10
	AG + GG	13180 ± 5035	58.49 ± 14.27	29.39 ± 13.85	34.33 ± 29.87	6.6 ± 6.13	23.21 ± 6.06	0.49 ± 0.09
	*p*	0.286^∗^	0.506^∗^	0.540^∗^	0.165^∗^	0.950^∗^	0.325^∗^	0.212^∗^
TLR4 Thr399Ile	CC	14678 ± 5805	56.27 ± 16.25	31.25 ± 14.45	45.76 ± 33.45	7.85 ± 6.5	22.28 ± 7.01	0.47 ± 0.10
	CT + TT	11720	34.3	56.3	-	0.39	23	0.53
	*p*	0.634^∗^	0.154^∗^	0.135^∗^	N/A	0.248^∗^	0.785^∗^	0.377^∗^

^∗^Kruskal-Wallis Test. WBC: white blood cells; ESR: erythrocyte sedimentation rate; CRP: C-reactive protein.

**Table 8:** Analysis of the relation between genotypes and immunoglobulins, IgG subclasses, C3, and C4.

SNPs	Genotypes	Mean ± SD (*n*)								
		IgG (g/L)	IgA (g/L)	IgM (g/L)	IgG1 (g/L)	IgG2 (g/L)	IgG3 (g/L)	IgG4 (g/L)	C3 (g/L)	C4 (g/L)
TLR4 Asp299Gly	AA	6.72 ± 3.00	0.54 ± 0.50	0.91 ± 0.44	4.82 ± 2.43	1.23 ± 0.70	0.27 ± 0.12	0.51 ± 0.40	1.43 ± 0.45	0.23 ± 0.07
	AG + GG	7.89 ± 3.51	0.76 ± 0.48	0.89 ± 0.48	5.14 ± 2.29	1.45 ± 0.96	0.35 ± 0.22	0.39 ± 0.34	1.52 ± 0.44	0.22 ± 0.05
	*p*	0.347^∗^	0.101^∗^	0.709^∗^	0.634^∗^	0.542^∗^	0.277^∗^	0.283^∗^	0.488^∗^	0.888^∗^
TLR4 Thr399Ile	CC	7.07 ± 3.17	0.60 ± 0.51	0.90 ± 0.45	4.93 ± 2.39	1.31 ± 0.78	0.29 ± 0.16	0.47 ± 0.40	1.42 ± 0.45	0.22 ± 0.06
	CT + TT	5.210	0.530	1.050	4.020	0.860	0.340	0.180	1.540	0.329
	*p*	0.572^∗^	0.944^∗^	0.572^∗^	0.735^∗^	0.735^∗^	0.598^∗^	0.970^∗^	0.590^∗^	0.190^∗^

^∗^Kruskal-Wallis Test.

## Data Availability

The data used to support the findings of this study are available from the corresponding author upon request.
